# Dynamic changes of atrioventricular conduction during Covid-19 infection: Does inflammation matter?

**DOI:** 10.1186/s42444-022-00072-0

**Published:** 2022-08-01

**Authors:** Risca Rini Aryanti, Dony Yugo Hermanto, Yoga Yuniadi

**Affiliations:** Department of Cardiology and Vascular Medicine, Faculty of Medicine, Universitas Indonesia and Arrhythmia Division, National Cardiovascular Center Harapan Kita, Jalan S Parman Kav 87 Slipi, Jakarta, 11420 Indonesia

**Keywords:** COVID-19, Inflammatory, Complete AV block, His bundle pacing

## Abstract

**Background:**

The primary manifestation of COVID-19 infection was pulmonary involvement. However, it can also manifest as a cardiovascular problem.

**Methods:**

We report a case of 82-year-old male COVID-19 patient who experienced atrioventricular (AV) conduction disturbance.

**Results:**

The rhythm was degenerated from sinus rhythm to complete AV block. We observe dynamic AV node dysfunction associated with inflammatory response. His bundle pacing successfully captured distal His region.

**Conclusion:**

The severe inflammatory response during COVID-19 infection might permanently damage cardiac conduction system resulted in a complete AV node block.

## Introduction

The global coronavirus disease 2019 (COVID-19) pandemic has impacted worldwide with mortalities of millions of cases. The primary manifestation of COVID-19 infection was pulmonary involvement. However, cardiovascular comorbidities were not uncommon in patients contracted with the virus ranging from 14 to 28% in several studies [1–3]. Arrhythmias is one of the cardiovascular manifestations; in one single cohort study of 390 hospitalized COVID-19 patients, the incidence of cardiac arrhythmias was 7.2%. Atrial fibrillation is becoming the most common case, while bradyarrhythmia only accounts for 10% of all arrhythmia [4]. We present a case of a COVID-19 patient with cardiovascular comorbidities who developed worsening atrioventricular (AV) block during hospitalization despite normal cardiac injury biomarkers.

## Case report

An 82-year-old male contracted with COVID-19 virus was brought to the hospital with complaints of cough and dyspnea. His previous medical history includes Non-ST Elevation Myocardial Infarction (NSTEMI) 4 years before admission, which were treated by drug-eluting stent placement. Left ventricular function was normal with a left ventricle ejection fraction (LVEF) of 69%. He was taking ramipril, spironolactone, bisoprolol, acetyl salicylic acid, and atorvastatin daily.

**Fig. 1:**
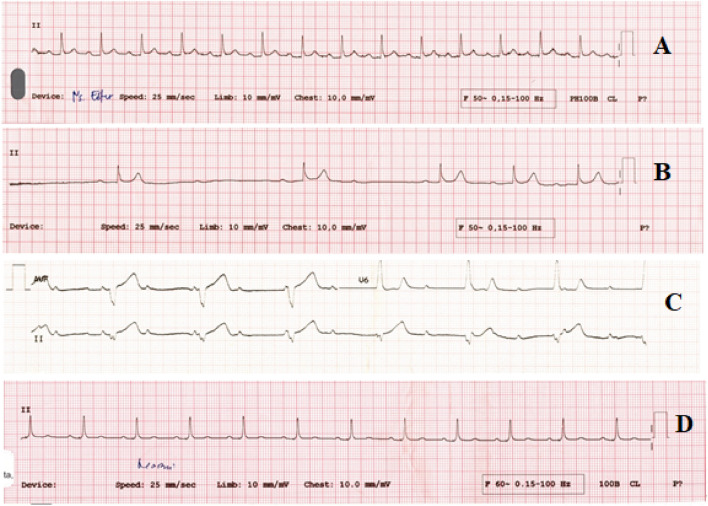
**A**. ECG at admission showed first degree AV block with narrow QRS duration, **B**. At second day hospitalization showed a 2:1 alternating with Mobitz 1 AV Block, **C**. On the 9th day of hospitalization, complete AV block with idioventricular escape rhythm occurred suggesting infra-His block, **D**. His bundle pacemaker implantation resulted in narrow QRS duration and pseudo-delta wave preceded with pacing spike resembling non-selective His bundle pacing

**Fig. 2:**
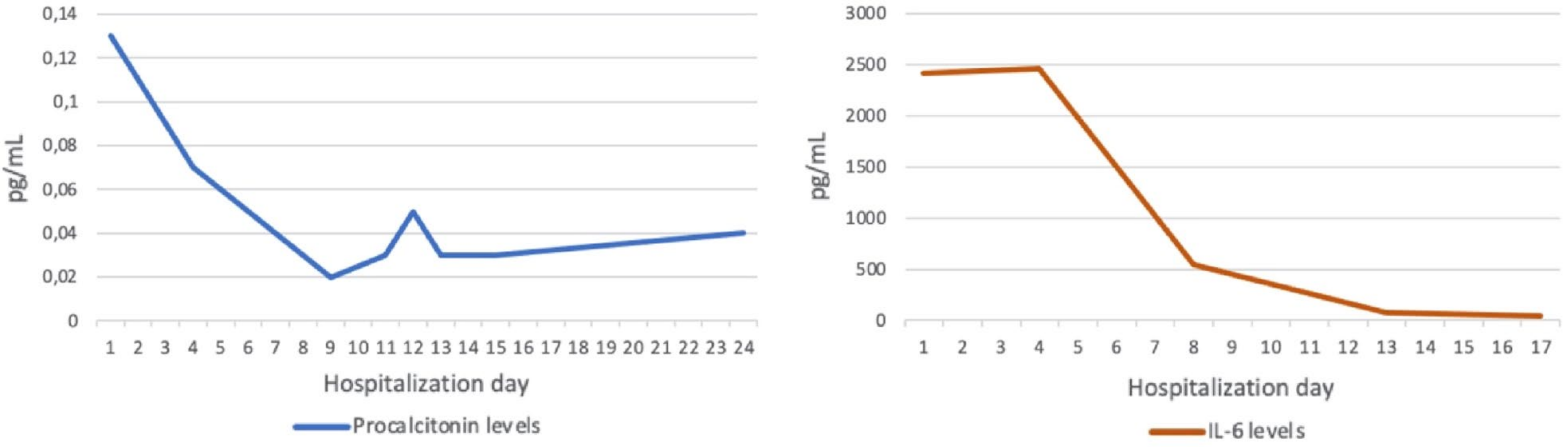
Inflammatory markers trend during hospitalization

Emergency department admission showed normal vital signs with a normal physical examination. Peripheral oximetry showed blood oxygen saturation of 96%. ECG showed first degree AV block with a rate of 86 bpm and PR interval of 240 ms (Fig. 1A). Slight cardiomegaly and ground-glass opacities were found in his chest X-ray. Potassium was normal with borderline kidney function (serum creatinine 0.55 mg/dL), while inflammatory markers were elevated (procalcitonin 0.13 ng/dL, CRP 56 mg/dL, and IL-6 135.6 pg/dL). On the second day of hospitalization, he felt general weakness with blood pressure fell to 70/30 mmHg. The rhythm was changed to a 2:1 AV block with a rate of 63 bpm (Fig. 1B). Serial high sensitivity cardiac troponin T (hs-cTnT) showed no increment (41 ng/dL to 46 ng/dL), CKMB 13 U/L***,*** while NT-pro-BNP was not elevated (724 pg/mL) according to his age. Intravenous fluid could resolve his hypotension, and a dopamine infusion of 5 mcg/kg/min was given. From day 3 to day 9 hospitalization, the rhythm was interchangeable between first degree AV block, Mobitz type 1 and Mobitz type 2 block. On the ninth day of hospitalization, the rhythm degenerated to complete AV block with a ventricle escape rhythm of 36 bpm (Fig. 1C). At that time, the level of IL-6 was high 2417 pg/mL and increased to 2457 pg/mL three days later. Due to hemodynamic compromise, we inserted a transvenous temporary pacemaker. A 2-week-period observation did not result in improvement of the AV block despite the IL-6 level dropped (Fig. 2). The patient was given tocilizumab 400 mg IV 2 times, dexamethasone 6 mg IV, remdesivir 200 mg IV for ten days during the observation period, and PCR swab test showed negative result at the 20th day of hospitalization. During hospitalization, the peripheral oxygen saturation ranging from 90 to 99%, in times of low oxygen levels, he received high flow nasal cannula therapy. Due to the permanent nature of the complete AV block, we decided to implant a dual chamber His bundle pacemaker. The ECG after implantation showed A sense and His Bundle pacing (Fig. 1D).

## Discussion

Having cardiovascular comorbidities worsen the prognosis in patients contracted with COVID-19 virus. A retrospective analysis revealed that during the pandemic, in-hospital mortality rate in patients admitted with cardiovascular disease was higher than before the pandemic (10.4% vs. 5.7%) [5]. Cardiac injury somewhat correlated with the increasing number of mortalities among patients with cardiovascular diseases. One cohort study showed that among 416 patients hospitalized for COVID-19, 19.7% of them had cardiac injury. Cardiac injury is defined as the elevation of cardiac biomarkers (hs-cTnT) above 99th percentile of upper limit range, regardless of the ECG and echocardiographic changes. A higher risk of mortality was expected in COVID-19 patients with cardiac injury compared to patients without cardiac injury [3]. The exact mechanism of cardiac injury in COVID-19 patients is still debatable. One of the accepted hypotheses is the direct invasion of the virus to myocardial cells by binding to angiotensin-converting enzyme-2 receptor (ACE-2) that was abundant in the heart. A meta-analysis study showed that cardiac injury risk was greater in patients with COVID-19 and other viruses that bind to ACE-2 than those that do not bind to ACE-2 receptors [6]. Other mechanisms that are likely involved in developing cardiac injury in COVID-19 patients are hypoxemia, severe inflammatory response syndrome, arteriovenous thrombosis, and coronary vasospasm [6, 7]. The risk of cardiac injury was higher in patients with older age, more comorbidities (e.g., hypertension), higher leukocyte counts, C-reactive protein levels, procalcitonin, N-terminal pro-B-type, creatinine, and had a higher proportion of multiple mottling and ground-glass opacity in radiographic findings [8, 9]. Our patient had some of the risks mentioned above, so he was susceptible to cardiac injury. However, our patient did not show any evidence of cardiac troponins and cardiac biomarkers elevation during hospitalization. The ECG did not show any ST T changes and elevated cardiac enzyme; therefore, we did not perform coronary angiography to the patient.

The hallmark of this case was the development of progressive AV block that occurred at the level of supraHis (narrow QRS complex) and spread to infra-His region (wide QRS complex) without any evidence of overt cardiac injury. At first, we suspect the high vagal tone played a role in developing the Mobitz type 1 block that occurred early in this patient. However, the irreversible nature of the block does not support that notion. Two case reports also described new complete AV block during hospitalization of COVID-19 patients, which resulted in permanent pacemaker implantation [10, 11]. Meanwhile, another case series reported 4 COVID-19 patients who developed transient AV block [12]. Several mechanisms could play roles in this setting. Cytokine storm could induce a chain reaction that causing myocardial ischemia, which affects AV node function transiently [13]. This notion may not be suited for our patient that developed a permanent AV node dysfunction. However, cytokine storm and SIRS could induce an inflammatory response that acted directly on cardiac ion channels causing AV node dysfunction [14]. Our patient was likely to have a cytokine storm due to the high IL-6 and CRP levels. In an animal study, it was found that IL-6 significantly reduced connexin 40 and connexin 43 expression in HL‐1 mouse atrial myocytes [15]. Connexins expressions are abundant in the AV node, and there are four different isoforms of connexins contained in the AV node. It was postulated that impaired AV node conduction could be related to the downregulation of connexins expression [16]. At ninth day of hospitalization when the complete AV block happened, procalcitonin level and IL-6 level remain high. Direct invasion of the virus is also possible due to the expression of ACE-2 receptors in the AV node region [17]. This also can cause irreversible damage to the AV node. Moreover, the exact mechanism of AV node injury in patients with COVID-19 is still unclear. Further studies regarding this matter are needed to predict the reversibility course of the block that could lead to better timing of permanent pacemaker implantation. Interestingly, pacing of the distal His bundle successfully captured it suggesting inflammation mediated block is limited to the proximal His bundle region. Sharma et al. [17] nicely showed a proposed model of longitudinal dissociation in the His bundle where fibers predestined for either the right bundle branch (RBB) or the left bundle branch (LBB). The inflammation or disease resulting in a delay in LBB conduction. His pacing lead position at distal of blocking site maintain normal conduction via native Purkinje system.

## Conclusion

Cardiac complications of COVID-19 may include a permanent high degree AV block requiring a permanent pacemaker implantation. The excessive inflammatory response may play a major role, but further researches are needed to explore the exact underlying mechanism.

## Data Availability

All supporting data for publication have been attached to the manuscript. If additional data are needed, we are happy to provide it.

## Abbreviations

COVID-19: Coronavirus disease 2019
NSTEMI: Non-ST Elevation Myocardial Infarction
AV Block: Atrioventricular Node Block
LVEF: Left ventricle ejection fraction
ECG: Electrocardiography
CRP: C-reactive protein
CKMB: Creatinine Kinase Myocardial Band
NT-pro-BNP: N-terminal prohormone of Brain Natriuretic Peptide
ACE-2: Angiotensin-converting enzyme-2
IL-6: Interleukin 6
